# Association of blood essential and non-essential element levels with echocardiographic parameters among Saudi heart failure patients: a case-control study

**DOI:** 10.3389/fcvm.2026.1743045

**Published:** 2026-04-17

**Authors:** Samah F. Ibrahim, Maha A. Al-Mohaissen, Terry Lee, Ahmed S. Gouhar, Mohammed N. Janjua, Nada Alhafez, Faisal Alghamdi, Shahzad Saeed, Halah z. Al-Rawi, Elaf S. Altuwalah

**Affiliations:** 1 Department of Internal Medicine, College of Medicine, Princess Nourah Bint Abdulrahman University, Riyadh, Saudi Arabia; 2 Centre for Advancing Health Outcomes, Vancouver, BC, Canada; 3 Training Deputyship, Naif Arab University for Security Sciences, Riyadh, Saudi Arabia; 4 Department of Forensic Sciences, College of Criminal Justice, Naif Arab University for Security Sciences, Riyadh, Saudi Arabia; 5 Cardiology Department, King Fahad Medical City, Riyadh Second Cluster, Riyadh, Saudi Arabia; 6 Research Center, King Fahad Medical City, Riyadh Second Cluster, Riyadh, Saudi Arabia; 7 College of Science, Princess Nourah Bint Abdulrahman University, Riyadh, Saudi Arabia

**Keywords:** cadmium, chromium, echocardiography, heart failure, iron, manganese, zinc

## Abstract

**Background:**

Essential elements such as manganese (Mn), iron (Fe), and zinc (Zn) are critical in for cardiovascular function, whereas exposure to non-essential toxic metals, including cadmium (Cd) and chromium (Cr) may contribute to cardiac dysfunction and the development or progression of heart failure (HF).

**Aim:**

This study evaluated the blood levels of Mn, Fe, Zn, Cd, and Cr in adult Saudi patients with HF and in controls with normal ventricular function, and explored their associations with HF status, functional class, and echocardiographic parameters.

**Methods:**

Consecutive adult patients referred for clinically indicated transthoracic echocardiography at the outpatient department of King Fahad Medical City hospital between November 2019 and March 2020 were invited to participate. Clinical data were collected, echocardiograms were reviewed, and blood concentrations of of Mn, Fe, Zn, Cd, and Cr were measured using atomic absorption spectrophotometry.

**Result:**

A total of 180 participants were included (mean age 60.3 years), of whom 65.6% had HF. Most measured element levels were within reference ranges, except for Mn, which was below the reference range (<4 ug/L) in 98% of participants, with median (IQR) of 0.40 (0.30, 0.60). Higher Cr levels were observed among younger participants (*P* = 0.046). Higher Cd levels were associated with increased left ventricular dilatation (*P* = 0.013; *P* < 0.001 after adjusting for confounders). Conversly, higher Mn levels were associated with lower lateral mitral annular velocity in univariate analysis (*P* = 0.04), but this association was not retained after multivariable adjustment (*P* = 0.099).

**Conclusion:**

This study provides insight into Mn, Fe, Zn, Cd, and Cr concentrations across a spectrum of HF severity. Alteration in Cd and Mn blood levels were associated with selected echocardiographic markers of HF severity. Further longitudinal studies are needed to establish the prognostic significance of element imbalances in HF.

## Introduction

Heart failure (HF) is a major and growing public health challenge worldwide (1). In the Middle East, including Saudi Arabia, the burden of HF is substantial, with an estimated 3.75 million affected individuals. This rising prevalence parallels increased industrialization, urbanization, and adoption of unhealthy lifestyle behaviors, which collectively contribute to the growing burden of cardiovascular risk factors (2).

In recent years, attention has focused on the role of environmental and dietary exposures in cardiovascular disease. Populations in the Middle East are increasingly exposed to essential and non-essential elements through air, water, food, and manufactured products. While essential trace elements are required for normal physiological processes, both deficiencies and excesses may adversely affect cardiovascular health (3). Conversely, non-essential or toxic elements may exert harmful effects, including cardiac disease, even at low concentrations (4, 5).

Essential trace elements such as manganese (Mn) (6, 7), iron (Fe), and zinc (Zn) (8) play critical roles in cardiovascular physiology by supporting antioxidant defense systems, mitochondrial function, and intracellular signaling pathways. However, its imbalance has been linked to an increased risk of cardiovascular disease, as they can impair cardiac performance and promote maladaptive responses in HF (9).

Fe is responsible for oxygen transport, mitochondrial respiration, and enzymatic activity (10). Its deficiency has been linked to impaired myocardial energetics and adverse outcomes in HF (11). As a result, its blood concentrations should be kept between 4,920–10,040 ug/L for women and 5,420–10,320 ug/L for men (12). Zinc is plays multiple significant physiological roles such as catalytic, structural, and regulatory roles. It regulates cardiac homeostasis through regulation of oxidative stress, calcium signaling, and inflammatory pathways (13, 14). Low Zn levels have been associated with poor prognosis in cardiovascular disease. Therefore, its concentrations should be kept between 80 and 120 ug/dL (8, 15). Mn acts as a cofactor for metabolism, energy production, and connective tissue growth. It acts as antioxidant enzymes cofactor, particularly mitochondrial superoxide dismutase, and contributes to vascular protection and metabolic regulation. Manganese intoxication raises significant health concerns, particularly neurotoxicity by enhancing mitochondrial dysfunction, oxidative stress, and apoptosis (14, 16),. Therefore, its levels should be maintained between 0.4–0.85 ug/L (7).

In contrast, non-essential elements such as cadmium (Cd) and chromium (Cr) may have deleterious cardiovascular effects even at low concentrations (14). Cadmium, commonly encountered through tobacco smoke and environmental contamination, accumulates in tissues. The elevated blood Cd levels; more than 5.0 ng/mL; has been implicated in oxidative stress, endothelial dysfunction, atherosclerosis, and myocardial injury. Its damage is linked to initiation of vascular inflammation, inhibition of cellular proliferation, and the induction of DNA damage, and apoptotic signaling pathway (14). Chromium, once considered an essential element, is now regarded as a cofactor modulating insulin action (17), with emerging evidence suggesting that excessive exposure—particularly to hexavalent forms—may contribute to various health hazards, including cancer, hematological and cardiovascular diseases (18–20). Therefore, its concentrations should be kept at or below ≤1.4 ug/L (21).

Although numerous studies have examined essential and non-essential elements disturbances in cardiovascular disease (8, 11, 14, 18, 19, 22–24), findings remain inconsistent and population-specific. Given the influence of dietary habits, genetic factors, and environmental exposures on element status, population-based biomonitoring is needed to clarify these relationships and enhance cardiovascular health. Therefore, this study aimed to evaluate blood levels of Mn, Fe, Zn, Cd, and Cr in adult Saudi participants with and without HF and to examine their associations with HF status, NYHA functional class, and echocardiographic indices of disease severity. The selection of these elements was hypothesis-driven, based on prior evidence linking them to myocardial metabolism, oxidative stress, and environmentally relevant toxic exposure. Other trace elements, such as selenium and copper, although biologically important, were beyond the scope of the present analysis to maintain analytical focus and avoid dilution of statistical power.

## Materials and methods

### Study design

This was a case-control study conducted at the outpatient echocardiography department of King Fahed Medical City, Riyadh, Saudi Arabia, between January 2019 and March 2020. The study protocol was approved by institutional review board (reference number: 19–192E), and written informed consent was obtained from all participants. Sample size calculation using G-power with a margin of error of 5%, level of confidence 95% and statistical power of 80% indicated minimum required sample of 160 participants.

### Study participants and echocardiographic assessments

A convenient sample of adult men and women (≥18 years) referred for clinically indicated echocardiography were included in the study. Exclusion criteria included connective tissue disease, vasculitis, infiltrative diseases (sarcoidosis, amyloidosis, hemochromatosis), hypertrophic cardiomyopathy, and pregnancy or postpartum status. Severe renal impairment (estimated glomerular filtration rate <30 mL/min/1.73 m^2^ or a serum creatinine level ≥2.5 mg/dL) was also excluded to minimize measurement bias and to ensure that measured element concentrations more accurately reflected true exposure and physiological status (25).

Echocardiographic examinations were performed by trained sonographers blinded to participants' clinical and biochemical data, using GE system Vivid 3 echocardiography machine. Left ventricular (LV) volume and LV ejection fraction (LVEF), which were evaluated using the biplane method of discs (26), in addition to the ratio of early to late ventricular filling velocities (E/A ratio), early diastolic mitral annular tissue velocities (setal, lateral, and average é), E to average é (E/é) ratio, and peak velocity of the tricuspid regurgitation jet, which were used for evaluation of LV diastolic function (27). HF classification was based on clinical assessment and echocardiographic findings and categorized as HF with reduced ejection fraction (HFrEF: LVEF was ≤40%); HF with mildly reduced ejection fraction (HFmrEF: LVEF was 41%–49%); and HF with preserved ejection fraction (HFpEF: LVEF ≥50% with evidence of diastolic dysfunction) (28, 29).

Control participants aged more than 18 years old were recruited during the same period from the echocardiography department after undergoing clinically indicated echocardiograms that revealed normal systolic and diastolic function without significant valvular disease (no pathological valve disease on 2D echocardiography and doppler assessment (except for grade 1 physiological regurgitation and mild age-related sclerosis) (30). The final sample size comprised of 180 participants.

### Blood samples and trace element analysis

Blood samples were collected into heparinized tubes. According to the method mentioned by Ahmed et al. (31), 750 µL of the whole blood sample was transferred to pre-acid-washed polytetrafluoroethylene (PTFE) microwave tube with 1.5 mL of concentrated nitric acid (69%) containing 200 µL of hydrogen peroxide (30%). Samples were incubated for 10 min, then 800 µL extra pure deionized water were added. The PTEF tubes were firmly closed and loaded into two steps microwave digestion program; 15 and 35 min, respectively with 1,500 watts power at 150°C temperature. The disgusted sample was transferred to flasks and nitric acid (2%) was added to obtain 500 µL mixture to perform ready analyzed sample that was preserved in pre-acid-washed PTFE tube at 23–25°C. Elements concentrations were measured in these samples using inductively coupled plasma optical emission spectrometry (model 5,110, Agilent Technologies, USA) according to the manufacturer's instructions.

### Statistical analysis

Statistical analysis was performed using SPSS software package V.20 (California, USA). Categorical data of descriptive analysis was presented as frequency (and percent) and continuous data was presented as mean ± standard deviation (SD) or median (interquartile range). Chi-square test was applied for comparison of categorical data between HF patients and healthy group. For continuous data, analysis of variance or Kruskal–Wallis test was applied as appropriate. To compare essential/non-essential element concentrations between groups defined by patient characteristics and echocardiographic parameters, Kruskal–Wallis test was used. Significance level was set at *P* < 0.05. Correction for multiple comparisons was not considered given the exploratory nature of the study.

## Results

A total of 180 participants were included; 118 (65.6%) HF patients and 62 (34.4%) controls. The mean participants age was 60.3 ± 12 years and 68.3% were male. Approximately, 69.4% of the participants had hypertension, 60% had diabetes, 66.1% had dyslipidemia, and 47.2% were present or past smokers. Of the patients with HF, 53.4% (*n* = 63) had HFrEF, 21.2% (*n* = 25) had HFmEF, and 18.6% (*n* = 22) had HFpEF Table 1.

**Table 1:** Baseline participants’ characteristics and cardiovascular risk factors comparison.

Column *N* (%) Variable	All 180	Normal (*N* = 62)	Heart failure with ejection altered ejection fraction 118 (100)				*P*
			Mildly reduced	Reduced	Preserved	Indeterminate	
Number (%)	180 (100)	62 (100)	25 (21.2)	63 (53.4)	22 (18.6)	8 (6.8)	
Age							0.001*
Mean (SD)	60.3 (12.0)	59.0 (9.5)	58.5 (14.6)	58.2 (13.1)	68.7 (8.5)	68.2 (8.8)	
Gender							˂0.001*
Male	123 (68.3)	39 (62.9)	18 (72.0)	55 (87.3)	7 (31.8)	4 (50.0)	
Female	57 (31.7)	23 (37.1)	7 (28.0)	8 (12.7)	15 (68.2)	4 (50.0)	
Hypertension	125 (69.4)	39 (62.9)	14 (56.0)	48 (76.2)	16 (72.7)	8 (100.0)	0.079
Diabetes mellitus	108 (60.0)	30 (48.4)	15 (60.0)	40 (63.5)	16 (72.7)	7 (87.5)	0.099
Dyslipidemia	119 (66.1)	38 (61.3)	15 (60.0)	44 (69.8)	16 (72.7)	6 (75.0)	0.706
Smoking							0.001*
No	95 (52.8)	30 (48.4)	10 (40.0)	28 (44.4)	20 (90.9)	7 (87.5)	
Yes cigarette	55 (30.6)	21 (33.9)	8 (32.0)	24 (38.1)	1 (4.5)	1 (12.5)	
Yes Hookah	7 (3.9)	3 (4.8)	1 (4.0)	3 (4.8)	0 (0.0)	0 (0.0)	
X-smoker	10 (5.6)	0 (0.0)	4 (16.0)	6 (9.5)	0 (0.0)	0 (0.0)	
Passive smoker	13 (7.2)	8 (12.9)	2 (8.0)	2 (3.2)	1 (4.5)	0 (0.0)	
Body mass index							0.041*
Underweight/Normal	42 (23.3)	15 (24.2)	10 (40.0)	14 (22.2)	3 (13.6)	0 (0.0)	
Overweight	50 (27.8)	19 (30.6)	4 (16.0)	22 (34.9)	2 (9.1)	3 (37.5)	
Obesity	88 (48.9)	28 (45.2)	11 (44.0)	27 (42.9)	17 (77.3)	5 (62.5)	

* Significant *P*-value ˂0.05.

The cardiovascular risk factors were more prevalent among HF participants. Regarding patient with HFpEF, 68.2% were female, 72.7% had hypertension, diabetes, and hyperlipidemia, 90.9% were non-smokers, and 86.4% were overweight or obese. Conversely, of the participants with HFrEF, 87.3% were male, 76.2% had hypertension, 63.5% had diabetes, and 69.8% had hyperlipidemia, 55.6% were smokers, and 77.8% were overweight or obese. According to Chi-square test result, the frequencies of risk factors such as age (P; 0.001), male gender (*P*;<0.001), smoking (P;0.001), and obesity (P;0.041) were statistically higher in HF participants (*P* < 0.05) (Table 1).

Median (IQR) concentrations of cadmium, chromium, manganese, iron, and zinc were 0.12 (0.07, 0.25) ng/mL, 1.61 (0.50, 2.12) ug/L, 0.40 (0.30, 0.60) ug/L, 7,411.9 (6,434.6, 8,192.5) ug/L, and 98.4 (83.6, 112.7) ug/dL, respectively. Nearly all participants (98%) had manganese levels below 4 ug/L, the reference range; whereas most other elements were within normal limits (Table 2).

**Table 2:** Blood trace elements concentrations among participants.

Variable	All 180 Column *n* (%)	Normal 62 Row *n* (%)	Heart failure 118 Row *n* (%)	*P*-value
Cadmium				
Median (IQR)	0.12 (0.07, 0.25)	0.13 (0.06, 0.23)	0.13 (0.07, 0.26)	0.611
<0.5 ng/mL, *n* (%)	175 (97.2)	60 (34.3)	115 (65.7)	
≥0.5 ng/mL, *n* (%)	5 (2.8)	2 (40)	3 (60)	
Chromium				
Median (IQR)	1.61 (0.50, 2.12)	1.63 (0.53, 2.15)	1.60 (0.48, 2.07)	0.629
≤1.4 ug/L, *n* (%)	80 (44.4)	28 (35.9)	50 (64.1)	
>1.4 ug/L, *n* (%)	100 (55.6)	34 (36.2)	60 (63.8)	
Manganese				
Median (IQR)	0.40 (0.30, 0.60)	0.36 (0.29, 0.60)	0.41 (0.30, 0.53)	0.304
<4 ug/L, *n* (%)	177 (98.3)	61 (34.5)	116 (65.5)	
4 to 15 ug/L, *n* (%)	2 (1.1)	1 (50)	1 (50)	
>15 ug/L, *n* (%)	1 (0.56)		1 (100)	
Iron				
Median (IQR)	7,411.9 (6,434.6, 8,192.5)	7,488.4 (6,507.9, 8,236.5)	7,397.9 (6,434.7, 8,161.8)	0.401
Women < 4,920, Men < 5,420 ug/L, *n* (%)	19 (10.6)	3 (16.7)	15 (83.3)	
Women: 4,920–10,040, Men 5,420–10,320 ug/L, *n* (%)	152 (84.4)	54 (37.2)	91 (62.8)	
Women > 10,040, Men > 10,320 ug/L, *n* (%)	9 (5)	5 (55.6)	4 (44.4)	
Zinc				
Median (IQR)	98.4 (83.6, 112.7)	101.5 (84.9, 118.7)	97.5 (80.7, 111.1)	0.087
<80 ug/dL, *n* (%)	34 (18.9)	9 (26.5)	25 (73.5)	
80 to 120 ug/dL, *n* (%)	116 (64.4)	39 (35.8)	70 (64.2)	
>120 ug/dL, *n* (%)	30 (16.7)	14 (48.3)	15 (51.7)	

*n* (%); number (percentage), Reference value of cadmium (<5.0 ng/mL), chromium(≤1.4 ug/L), manganese (4–15 ug/L), iron (4,920–10,040 ug/L for women and 5,420–10,320 ug/L for men), and zinc (80–120 ug/dL).

Regarding age, there was a general tendency for the tested elements levels to decrease with age, except for manganese showed stable levels. Regarding gender, women had higher levels of chromium, iron, and zinc**.** Chronic health status was associated with changes in the different elements' levels. For example, hypertensive and obese individuals had increased levels of iron, and zinc; diabetic individuals had higher levels of chromium; and participants with dyslipidemia showed increased levels of manganese, and zinc. In addition, zinc was found in higher concentrations in the blood samples of smokers, while cadmium and chromium levels were higher in passive smokers. Our results showed significantly higher concentrations of chromium among younger participants (<40: 2.12 (0.73, 2.98) vs. 40–59: 1.78 (0.54, 2.18) vs. ≥60: [148 (0.48, 2.02), *P* = 0.046], and significantly lower concentrations of cadmium among participants with dyslipidemia [no: 0.18 (0.10, 0.27) vs. yes: 0.12 (0.06, 0.20), *P* = 0.016]. However, our study did not observe any significant association between the levels of other elements and the cardiovascular risk factors (Table 3).

**Table 3:** Trace elements concentrations and its relation to participants’ characteristics and cardiovascular risk factors.

Variable		Cadmium		Chromium		Manganese		Iron		Zinc	
	N	Median (IQR)	*P*	Median (IQR)	*P*	Median (IQR)	*P*	Median (IQR)	*P*	Median (IQR)	*P*
Age			0.18		0.046*		0.096		0.203		0.264
<40	12	0.24 (0.08, 0.38)		2.12 (0.73, 2.98)		0.39 (0.28, 0.72)		8,016.2 (6,860.9, 9,487.1)		108.7 (89.3, 128.8)	
40–59	64	0.13 (0.08, 0.25)		1.78 (0.54, 2.18)		0.39 (0.30, 0.55)		7,579.2 (6,461.5, 8,220.6)		97.0 (80.3, 112.7)	
≥60	102	0.12 (0.05, 0.23)		1.48 (0.48, 2.02)		0.40 (0.30, 0.61)		7,358.9 (6,434.6, 8,027.6)		98.8 (83.9, 111.1)	
Sex			0.623		0.911		0.740		0.448		0.522
Male	123	0.12 (0.06, 0.24)		1.46 (0.48, 2.17)		0.40 (0.30, 0.59)		7,397.8 (6,299.3, 8,161.8)		97.4 (81.5, 115.4)	
Female	57	0.13 (0.07, 0.25)		1.65 (0.55, 2.02)		0.40 (0.29, 0.62)		7,417.8 (6,671.8, 8,196.2)		101.2 (86.0, 110.8)	
HTN			0.126		0.497		0.338		0.676		0.819
No	55	0.15 (0.08, 0.26)		1.65 (0.51, 2.19)		0.41 (0.31, 0.63)		7,280.8 (6,128.5, 8,236.5)		96.8 (80.0, 112.9)	
Yes	125	0.12 (0.05, 0.23)		1.60 (0.49, 2.08)		0.40 (0.29, 0.53)		7,417.7 (6,535.9, 8,067.0)		99.5 (84.1, 112.5)	
DM			0.382		0.730		0.490		0.151		0.584
No	72	0.13 (0.07, 0.26)		1.50 (0.48, 2.13)		0.42 (0.30, 0.58)		7,639.2 (6,400.7, 8,248.5)		98.6 (84.1, 115.2)	
Yes	108	0.12 (0.06, 0.24)		1.61 (0.51, 2.10)		0.39 (0.29, 0.60)		7,324.3 (6,435.1, 7,908.8)		98.4 (82.8, 111.8)	
Dyslipidemia			0.016*		0.874		0.725		0.562		0.919
No	61	0.18 (0.10, 0.27)		1.65 (0.56, 2.08)		0.37 (0.29, 0.60)		7,561.8 (6,416.0, 8,196.2)		97.6 (81.5, 116.4)	
Yes	119	0.12 (0.06, 0.20)		1.61 (0.49, 2.13)		0.40 (0.30, 0.53)		7,320.0 (6,434.7, 8,188.9)		99.0 (84.4, 111.3)	
Smoking			0.067		0.621		0.598		0.890		0.735
No	95	0.13 (0.05, 0.24)		1.61 (0.48, 2.09)		0.40 (0.30, 0.62)		7,407.8 (6,366.9, 8,072.6)		97.4 (83.8, 109.2)	
Yes cigarette	55	0.12 (0.07, 0.26)		1.60 (0.49, 2.13)		0.41 (0.33, 0.61)		7,417.7 (6,416.0, 8,518.3)		99.2 (81.3, 118.3)	
Yes Hookah	7	0.12 (0.10, 0.34)		0.90 (0.56, 2.50)		0.34 (0.28, 0.48)		8,068.0 (6,435.6, 8,629.4)		111.3 (81.5, 118.3)	
X-smoker	10	0.06 (0.04, 0.12)		1.94 (0.62, 2.50)		0.41 (0.28, 0.53)		7,719.5 (6,435.6, 8,417.8)		99.7 (92.5, 118.4)	
Passive smoker	13	0.20 (0.13, 0.25)		2.02 (0.54, 2.39)		0.33 (0.26, 0.60)		7,320.0 (6,887.3, 8,076.1)		94.7 (88.6, 116.3)	
BMI			0.649		0.505		0.667		0.465		0.661
Underweight/Normal	42	0.14 (0.08, 0.26)		1.69 (0.52, 2.19)		0.41 (0.30, 0.63)		7,238.5 (6,008.3, 8,072.6)		96.0 (81.5, 106.8)	
Overweight	50	0.12 (0.05, 0.26)		0.87 (0.45, 2.12)		0.40 (0.30, 0.56)		7,399.3 (6,535.9, 8,401.0)		95.7 (82.3, 116.3)	
Obesity	88	0.12 (0.06, 0.23)		1.61 (0.53, 2.09)		0.38 (0.29, 0.57)		7,417.8 (6,541.8, 8,192.5)		100.2 (84.2, 114.0)	

* Significant *P*-value ˂0.05.

Although element concentrations appeared to increase across New York Heart Association (NYHA) functional classes, these trends did not reach statistical significance (Table 4). Regarding echocardiographic findings, higher cadmium levels were associated with left ventricular dilatation (normal: 0.11 (0.05,0.21) vs. mild: 0.14 (0.07, 0.26) vs. moderate: 0.19 (0.09, 0.36) vs. severe 0.26 (0.13, 0.29), *P* = 0.013), while higher manganese levels were significantly associated with lower lateral mitral annular velocity, a marker of diastolic dysfunction [<10: 0.41 (0.30, 0.62) vs. ≥10: 0.35 (0.26,0.50), *P* = 0.04]. After accounting for potential confounders (HF type, age, sex, hypertension, diabetes, dyslipidemia, smoking and BMI) using quantile regression, the association between cadmium and left ventricular dilatation remained significant (overall *P* < 0.001; estimated difference (95% CI): normal vs. severe: −0.18 (−0.26, −0.09), mild vs. severe: −0.13 (−0.21, −0.05), moderate vs. severe: −0.12 (−0.21, −0.03), while the association between manganese and lateral mitral annular velocity was only marginally significant [<10 vs. ≥10: 0.07 (−0.01, 0.15); *P* = 0.099]. No significant associations were observed between zinc or iron levels and echocardiographic parameters (Table 5).

**Table 4:** Trace elements concentrations and its relation to New York heart association (NYHA) functional status, and cardiac biomarkers.

		Cadmium		Chromium		Manganese		Iron		Zinc	
NYHA	*N*	Median (IQR)	*P*	Median (IQR)	*P*	Median (IQR)	*P*	Median (IQR)	*P*	Median (IQR)	*P*
0 + 1	124	0.13 (0.07, 0.24)	0.173	1.60 (0.50, 2.14)	0.279	0.40 (0.30, 0.55)	0.398	7,404.3 (6,391.0, 8,183.2)	0.483	98.0 (83.9, 113.4)	0.444
2	39	0.12 (0.04, 0.25)		1.61 (0.46, 2.02)		0.43 (0.30, 0.63)		7,417.8 (6,507.9, 8,188.9)		93.2 (81.3, 106.2)	
3	13	0.24 (0.12, 0.34)		1.81 (0.69, 2.25)		0.30 (0.29, 0.61)		7,437.8 (6,834.5, 8,236.6)		105.9 (84.9, 125.4)	
4	2	0.09 (0.07, 0.11)		2.22 (1.81, 2.62)		0.69 (0.65, 0.73)		5,808.1 (4,975.7, 6,640.5)		113.7 (77.5, 149.8)	

**Table 5:** Elements concentrations and its relation to echocardiographic parameters.

Parameter	*N*	Cadmium	*P*	Chromium	*P*	Manganese	*P*	Iron	*P*	Zinc	*P*
LV Diagnosis			0.276		0.420		0.333		0.467		0.509
Normal	62	0.13 (0.06, 0.23)		1.63 (0.53, 2.15)		0.36 (0.29, 0.60)		7,488.4 (6,507.9, 8,236.5)		101.5 (84.9, 118.7)	
HFmrEF^a^	25	0.10 (0.07, 0.14)		1.77 (0.53, 2.33)		0.42 (0.33, 0.50)		7,117.2 (6,320.0, 7,823.9)		97.6 (91.9, 105.4)	
HFrEF^b^	63	0.15 (0.07, 0.27)		0.90 (0.44, 2.02)		0.40 (0.28, 0.50)		7,542.4 (6,435.6, 8,401.0)		93.7 (80.7, 112.3)	
HFpEF^c^	22	0.12 (0.04, 0.22)		1.71 (1.47, 2.10)		0.45 (0.35, 0.90)		7,348.4 (6,509.6, 7,683.6)		100.8 (83.5, 115.0)	
Indeterminate	8	0.10 (0.05, 0.13)		1.87 (1.13, 2.15)		0.38 (0.23, 0.54)		7,219.3 (5,666.6, 7,528.0)		97.4 (85.8, 106.4)	
LV volume			0.013*		0.733		0.908		0.391		0.829
Normal	128	0.11 (0.05, 0.21)		1.61 (0.54, 2.13)		0.40 (0.30, 0.63)		7,411.9 (6,333.1, 8,175.4)		99.1 (83.5, 113.4)	
Mildly dilated	31	0.14 (0.07, 0.26)		0.69 (0.42, 2.02)		0.38 (0.32, 0.50)		7,872.9 (6,727.3, 8,401.0)		97.4 (83.9, 116.3)	
Moderate dilated	8	0.19 (0.09, 0.36)		1.84 (0.48, 2.28)		0.38 (0.21, 0.49)		7,623.5 (6,869.3, 7,872.4)		93.6 (84.4, 104.5)	
Severely dilated	13	0.26 (0.13, 0.29)		1.17 (0.47, 2.01)		0.40 (0.29, 0.49)		7,117.1 (6,299.2, 8,067.0)		93.2 (83.9, 116.4)	
Lateral é			0.901		0.400		0.040*		0.832		0.650
<10	127	0.12 (0.07, 0.24)		1.60 (0.49, 2.08)		0.41 (0.30, 0.62)		7,417.8 (6,434.7, 8,204.5)		99.2 (83.4, 115.0)	
≥10	48	0.13 (0.05, 0.25)		1.81 (0.54, 2.21)		0.35 (0.26, 0.50)		7,304.7 (6,424.9, 8,076.1)		94.0 (83.1, 111.8)	
Septal é			0.396		0.263		0.258		0.329		0.671
<7	131	0.12 (0.07, 0.24)		1.60 (0.48, 2.08)		0.40 (0.30, 0.60)		7,397.9 (6,416.0, 8,188.9)		99.0 (82.3, 112.2)	
≥7	49	0.14 (0.06, 0.28)		1.81 (0.56, 2.22)		0.35 (0.29, 0.56)		7,561.8 (6,507.9, 8,204.5)		96.6 (84.9, 113.9)	
E/A ratio			0.135		0.859		0.634		0.831		0.963
≤0.8	40	0.13 (0.08, 0.23)		1.46 (0.47, 2.13)		0.40 (0.29, 0.55)		7,488.4 (6,683.9, 8,175.4)		97.7 (81.1, 119.7)	
>0.8 and <2	106	0.12 (0.05, 0.24)		1.61 (0.53, 2.10)		0.40 (0.30, 0.62)		7,417.7 (6,366.8, 8,236.5)		100.2 (85.7, 112.5)	
≥2	19	0.23 (0.12, 0.27)		1.17 (0.46, 2.03)		0.35 (0.28, 0.60)		7,060.4 (6,727.3, 7,823.9)		99.5 (82.1, 112.3)	
E/Av.e			0.446		0.585		0.080		0.202		0.690
<14	112	0.12 (0.06, 0.24)		1.63 (0.49, 2.15)		0.37 (0.28, 0.53)		7,475.6 (6,471.8, 8,220.6)		99.4 (82.8, 114.6)	
≥14	61	0.13 (0.07, 0.26)		1.60 (0.50, 2.01)		0.42 (0.32, 0.62)		7,117.2 (6,434.7, 7,872.9)		98.0 (85.7, 106.2)	
Diastolic dysfunction grade			0.442		0.891		0.145		0.704		0.764
0	56	0.13 (0.06, 0.23)		1.72 (0.53, 2.17)		0.34 (0.29, 0.60)		7,550.5 (6,392.3, 8,236.6)		100.1 (84.9, 117.0)	
1	47	0.13 (0.07, 0.25)		1.35 (0.43, 2.08)		0.43 (0.35, 0.52)		7,417.7 (6,435.6, 8,417.8)		97.6 (81.5, 112.2)	
2	36	0.12 (0.07, 0.27)		1.61 (0.62, 1.97)		0.43 (0.31, 0.82)		7,348.4 (6,343.4, 7,962.1)		100.8 (86.1, 115.2)	
3	18	0.19 (0.12, 0.27)		0.96 (0.46, 2.03)		0.38 (0.29, 0.60)		7,145.6 (6,727.3, 7,823.9)		102.4 (83.9, 112.3)	

^a^ Heart failure with mildly reduced ejection fraction.

^b^ Heart failure with reduced ejection fraction.

^c^ Heart failure with preserved ejection fraction Lateral é: Lateral mitral é annular velocity, Septal é: septal mitral é annular velocity, E/A ratio: the ratio of early to late ventricular filling velocities.

* Significant *P*-value < 0.05.

## Discussion

In the current Saudi study blood levels of cadmium, chromium, iron, and zinc were largely within the normal ranges, whereas manganese levels were markedly low in most participants. Variations in cadmium and chromium levels showed a tendency to be associated with established cardiovascular risk factors, including age, and dyslipidemia. Moreover, alteration of cadmium, and manganese levels were significantly associated with some echocardiographic parameters.

Alrobaian and Arida, (32) found that blood levels of cadmium, manganese, chromium, and zinc in Saudi population were almost below the reference values and the international tolerance values as well. Their findings also support our observation that younger smokers exhibited higher levels of the elements compared with older nonsmokers (32). Element concentrations are influenced by multiple factors, including procedural and measurement-related variability as well as biological determinants such as age, nutritional status, genetic background, lifestyle habits, and environmental exposures (3, 14).

Low manganese levels may reflect dietary insufficiency, as Mohamed et al. (33) showed that manganese intake from commonly consumed staple foods in Saudi Arabia contributes only for only about 17% of recommended intake (33). In addition, Genetic variability in manganese transport and homeostasis, such as SLC39A8/ZIP8 and SLC30A10, can also influence circulating levels and necessitates further investigation (34). Furthermore, Štiglic et al. (35) emphasized the importance of establishing population- and laboratory-specific reference ranges for essential and non-essential elements to ensure accurate clinical interpretation and strengthen biomonitoring and public health research (35).

Additionally, element concentrations varied according to age, sex, and comorbidities, with higher chromium, iron, and zinc levels observed in women; elevated zinc and iron in hypertensive and obese participants; increased chromium in individuals with diabetes; and higher cadmium, manganese and zinc in those with dyslipidemia. These variations likely reflect differences in dietary intake, supplement use, and age-related declines in absorption efficiency, contributing to the observed patterns in the studied populations (36, 37). Previous studies reported divergent findings regarding zinc and iron status across populations. Elevated zinc and iron levels among Nigerian population had been associated with hypertension through oxidative and atherogenic enhancement mechanisms (38), whereas zinc deficiency in Saudi patients with diabetes has been linked to adverse metabolic and cardiovascular outcomes (39). These discrepancies likely reflect differences in population characteristics, study design, and assessment methods, particularly given that iron and zinc levels in our Saudi cohort were within normal reference ranges.

In the present study, zinc and iron levels were not associated with clinical, laboratory, or echocardiographic parameters in HF patients. This contrasts with previous studies reporting adverse outcomes associated with zinc deficiency and iron deficiency in specific HF populations, particularly those with anemia or concomitant kidney disease, where supplementation had been shown to reduce NT-proBNP levels, improve NYHA functional class, and diastolic indices, resulting in improving cardiac function and reducing mortality outcome (23). These findings elaborates zinc's role in maintaining myocardial redox balance, enhancing antioxidant defense mechanisms, and attenuating inflammatory signaling with resulting apoptosis (8, 13, 15). Similarly, Toblli et al. (40) reported that iron supplementation in anemic patients with HF and concomitant kidney disease improved left ventricular performance, NYHA functional status, and neurohormonal markers (40) through favorable cardiac remodeling through attenuation of oxidative stress, inflammatory marker expression, hypoxia-related fibrosis, and hypertrophy. These discrepancies may reflect differences in patient characteristics, particularly anemia status, disease severity, and comorbidity burden (11, 41, 42).

Chromium exhibits variable effects on human health. Despite its uncertain role in macronutrient metabolism, excessive chromium exposure can accumulat in multiple organs, including the heart, where it may induce oxidative stress, inflammatory responses, and mitochondrial dysfunction, ultimately exacerbating cardiac pathology (43).

Manganese reveals a positive impact on cardiovascular health, through its antioxidant and atheroprotective effects, including inhibition of low-density lipoprotein oxidation, modulation of oxidative stress, enhancement of high-density lipoprotein cholesterol, and reduction in hypertension, metabolic syndrome, and abdominal obesity (16, 44–46). Previous studies identified an inverse association between serum manganese levels and cardiovascular disease risk (47, 48). However, excessive manganese exposure, particularly in occupational settings, has been associated with elevated myocardial enzymes and potential cardiovascular dysfunction, underscoring the need for balanced exposure (49). In the present study, higher manganese levels were significantly associated with decreased lateral mitral annular velocity in univariant analysis, a marker of diastolic dysfunction, suggesting a possible indirect influence of manganese status on cardiac performance. However, this difference was only marginally significant after adjusting for potential confounders. Collectively, both manganese deficiency and excess may adversely affect cardiovascular health, highlighting the need for further research to clarify optimal manganese levels and their echocardiographic correlates (50).

In contrast, cadmium demonstrated a consistently adverse cardiovascular profile. Blood cadmium concentrations were higher among passive smokers and increased with worsening New York Heart Association functional class, with higher levels significantly associated with left ventricular dilatation. Smoking is a major source of cadmium exposure and exerts a synergistic effect on systemic cadmium levels in both active and passive smokers (32, 51). The higher cadmium body burden linked to increased cardiovascular disease risk and mortality, even among non-smokers (52, 53).

Mechanistically, cadmium contributes to HF progression through induction of oxidative stress with lipid peroxidation, mitochondrial dysfunction, and cardiomyocyte injury, while disruption of calcium homeostasis impairs excitation–contraction coupling and promotes adverse ventricular remodeling, including left ventricular dilatation. In parallel, cadmium activates inflammatory pathways, endothelial dysfunction, and microvascular injury, thereby accelerating atherosclerotic processes. Renal accumulation of cadmium further augments proximal tubular damage, chronic kidney disease, and secondary hypertension, amplifying cardiovascular burden (52–56).

Consistent with our findings regarding the prevalence of HFpEF, Mancusi et al. (57) and Omar et al. (58) reported that HFpEF was more common among older adults, particularly women, and was frequently associated with comorbidities such as obesity, hypertension, diabetes, and atrial fibrillation (57, 58). The pathogenesis of HF has been linked to deficiencies in essential elements, including zinc, selenium, and magnesium, as well as imbalances in potentially toxic elements such as cadmium, manganese, lead, and copper. However, the complex interaction between environmental exposures and individual clinical characteristics may obscure these associations, underscoring the need for further targeted investigations to clarify these associations (59).

This study evaluated the blood levels of selected essential and non-essential/toxic elements among adult Saudi participants, including healthy controls and patients with HF. Several limitations should be acknowledged. Uncontrolled factors influencing element concentrations—such as dietary intake, environmental exposure, supplement use, medications, and biochemical interactions—limit causal inference. The single-center design and relatively small sample sizes within HF subgroups, particularly HFpEF, may reduce statistical power and generalizability. Also, given the exploratory nature of the study, we did not account for multiple comparisons in our analysis even though we examined a large number of parameters. In fact, none of our findings would remain statistically significant after false discovery rate correction. In addition, exclusion of hospitalized HF cases, who typically have more advanced disease, including renal impairment and reduced estimated glomerular filtration rate, may introduce selection bias, as the cohort primarily reflects clinically stable outpatients. Consequently, the findings may not be generalizable to patients with acutely decompensated or advanced HF, who also experience higher mortality rates (25, 60). Despite these limitations, the study provides insight into Mn, Fe, Zn, Cd, and Cr concentrations across a spectrum of HF severity, leading to a better understanding of the multifactorial nature of HF and informing potential preventive and therapeutic strategies. Future longitudinal studies with larger cohorts, well-controlled confounding factors, broader element panels, and mechanistic exploration are needed to clarify the role of element imbalances in HF progression across phenotypes and severity levels, including decompensated cases.

## Conclusion

Blood levels of the studied essential and non-essential elements were within normal ranges among both healthy participants and patients with HF, except for manganese, which was frequently low. While zinc and iron showed no association with clinical or echocardiographic parameters, variations in cadmium, chromium, and manganese were significantly associated with selected cardiovascular findings. These results suggest a potential role of specific elements in cardiovascular status among Saudi patients with HF that could be explored in future longitudinal and mechanistic studies.

## Data Availability

The raw data supporting the conclusions of this article will be made available by the authors, without undue reservation.

## References

[1] Daubert C. Heart failure: a major public health problem. Presse Med. (2024) 53(1):104224. 10.1016/j.lpm.2024.104224 38340897

[2] Elasfar AA Alhabeeb W Elasfar S. Heart failure in the Middle East Arab countries: current and future perspectives. J Saudi Heart Assoc. (2020) 32(2):236–41. 10.37616/2212-5043.1040 33154923 PMC7640557

[3] Rehman K Fatima F Waheed I Akash MSH. Prevalence of exposure of heavy metals and their impact on health consequences. J Cell Biochem. (2018) 119(1):157–84. 10.1002/jcb.26234 28643849

[4] Mitra S Chakraborty AJ Tareq AM Emran TB Nainu F Khusro A Impact of heavy metals on the environment and human health: novel therapeutic insights to counter the toxicity. J King Saud Univ Sci. (2022) 34(3):101865. 10.1016/j.jksus.2022.101865

[5] Demir F Kipcak AS Dere Ozdemir O Moroydor Derun E. Determination of essential and non-essential element concentrations and health risk assessment of some commercial fruit juices in Turkey. J Food Sci Technol. (2020) 57(12):4432–42. 10.1007/s13197-020-04480-9 33087957 PMC7550484

[6] Ross AC Caballero B Cousins RJ Tucker KL Ziegler TR. Modern Nutrition in Health and Disease. 11th ed. Burlington, MA: Jones & Bartlett Learning (2020).

[7] Williams M Daniel Todd G Roney N Crawford J Coles C McClure PR Toxicological Profile for Manganese. Atlanta (GA): Agency for Toxic Substances and Disease Registry (US) (2013). 24049862

[8] Knez M Glibetic M. Zinc as a biomarker of cardiovascular health. Front Nutr. (2021) 8:686078. 10.3389/fnut.2021.686078 34395491 PMC8360846

[9] Pan Z Gong T Liang P. Heavy metal exposure and cardiovascular disease. Circ Res. (2024) 134(9):1160–78. 10.1161/CIRCRESAHA.123.323617 38662861

[10] Zhang DL Ghosh MC Rouault TA. The physiological functions of iron regulatory proteins in iron homeostasis—an update. Front Pharmacol. (2014) 5:124. 10.3389/fphar.2014.00124 24982634 PMC4056636

[11] von Haehling S Ebner N Evertz R Ponikowski P Anker SD. Iron deficiency in heart failure. JACC Heart Fail. (2019) 7(1):36–46. 10.1016/j.jchf.2018.07.015 30553903

[12] BPD. *Iron: Reference Range, Interpretation, Collection and Panels*. *Medscape**.* (2025). Available online at: https://emedicine.medscape.com/article/2085704-overview (Accessed October 18, 2025).

[13] Kitala K Tanski D Godlewski J Krajewska-Włodarczyk M Gromadziński L Majewski M. Copper and zinc particles as regulators of cardiovascular system function—a review. Nutrients. (2023) 15(13):3040. 10.3390/nu15133040 37447366 PMC10346759

[14] Zoroddu MA Aaseth J Crisponi G Medici S Peana M Nurchi VM. The essential metals for humans: a brief overview. J Inorg Biochem. (2019) 195:120–9. 10.1016/j.jinorgbio.2019.03.013 30939379

[15] Hara T Yoshigai E Ohashi T Fukada T. Zinc in cardiovascular functions and diseases: epidemiology and molecular mechanisms for therapeutic development. Int J Mol Sci. (2023) 24(8):7152. 10.3390/ijms24087152 37108314 PMC10139119

[16] Chen L Deng H Cui H Fang J Zuo Z Deng J Inflammatory responses and inflammation-associated diseases in organs. Oncotarget. (2018) 9(6):7204–18. 10.18632/oncotarget.23208 29467962 PMC5805548

[17] Tsave O Yavropoulou MP Kafantari M Gabriel C Yovos JG Salifoglou A. The adipogenic potential of Cr(III). A molecular approach exemplifying metal-induced enhancement of insulin mimesis in diabetes mellitus II. J Inorg Biochem. (2016) 163:323–31. 10.1016/j.jinorgbio.2016.07.015 27633760

[18] Haider AM Ali A Saleh EAM Jalil AT Abdulelah FM Romero-Parra RM The role of chromium supplementation in cardiovascular risk factors: a comprehensive review of putative molecular mechanisms. Heliyon. (2023) 9(9):e19826. 10.1016/j.heliyon.2023.e19826 37809394 PMC10559203

[19] Sharma P Singh SP Parakh SK Tong YW. Health hazards of hexavalent chromium (Cr (VI)) and its microbial reduction. Bioengineered. (2022) 13(3):4923–38. 10.1080/21655979.2022.2037273 35164635 PMC8973695

[20] Vincent JB. New evidence against chromium as an essential trace element. J Nutr. (2017) 147(12):2212–9. 10.3945/jn.117.255901 29021369

[21] University of California, S.F. and Chromium—Blood Test. (2023). Available online at: https://www.ucsfhealth.org/medical-tests/chromium—blood-test (Accessed October 18, 2025).

[22] Guo Q Cai J Qu Q Cheang I Shi J Pang H Association of blood trace elements levels with cardiovascular disease in US adults: a cross-sectional study from the national health and nutrition examination survey 2011–2016. Biol Trace Elem Res. (2024) 202(7):3037–50. 10.1007/s12011-023-03913-8 37891364

[23] Alexanian I Parissis J Farmakis D Athanaselis S Pappas L Gavrielatos G Clinical and echocardiographic correlates of serum copper and zinc in acute and chronic heart failure. Clin Res Cardiol. (2014) 103:938–49. 10.1007/s00392-014-0735-x 24908339

[24] Mohammadifard N Humphries KH Gotay C Mena-Sánchez G Salas-Salvadó J Esmaillzadeh A Trace minerals intake: risks and benefits for cardiovascular health. Crit Rev Food Sci Nutr. (2019) 59(8):1334–46. 10.1080/10408398.2017.1406332 29236516

[25] Liu S Wang H Cao Y Lu L Wu Y Lian F The association between low-concentration heavy metal exposure and chronic kidney disease risk through *α*-klotho. Sci Rep. (2025) 15(1):11320. 10.1038/s41598-025-96016-4 40175481 PMC11965371

[26] Lang RM Badano LP Mor-Avi V Afilalo J Armstrong A Ernande L Recommendations for cardiac chamber quantification by echocardiography in adults: an update from the American society of echocardiography and the European association of cardiovascular imaging. Eur Heart J Cardiovasc Imaging. (2015) 16(3):233–71. 10.1093/ehjci/jev014 25712077

[27] Nagueh SF Smiseth OA Appleton CP Byrd BF Dokainish H Edvardsen T Recommendations for the evaluation of left ventricular diastolic function by echocardiography: an update from the American society of echocardiography and the European association of cardiovascular imaging. Eur J Echocardiogr. (2016) 17(12):1321–60. 10.1093/ehjci/jew082 27422899

[28] Swedberg K Cleland J Dargie H Drexler H Follath F Komajda M Guidelines for the diagnosis and treatment of chronic heart failure: executive summary (update 2005) the task force for the diagnosis and treatment of chronic heart failure of the European society of cardiology. Eur Heart J. (2005) 26(11):1115–40. 10.1093/eurheartj/ehi204 15901669

[29] Heidenreich PA Bozkurt B Aguilar D Allen LA Byun JJ Colvin MM 2022 AHA/ACC/HFSA guideline for the management of heart failure: a report of the American college of cardiology/American heart association joint committee on clinical practice guidelines. J Am Coll Cardiol. (2022) 79(17):e263–421. 10.1016/j.jacc.2021.12.012 35379503

[30] Rudski LG Lai WW Afilalo J Hua L Handschumacher MD Chandrasekaran K Guidelines for the echocardiographic assessment of the right heart in adults: a report from the American society of echocardiography endorsed by the European association of echocardiography, a registered branch of the European society of cardiology, and the Canadian society of echocardiography. J Am Soc Echocardiogr. (2010) 23(7):685–713; quiz 786–8. 10.1016/j.echo.2010.05.010 20620859

[31] Ahmed H Almohammed KMS Janjua MN Alhafez N. Metals determination in the whole blood by ICP-OES: a comparison of two digestion procedures. Int J Environ Anal Chem. (2024) 104(11):2642–55. 10.1080/03067319.2022.2070011

[32] Alrobaian M Arida H. Assessment of heavy and toxic metals in the blood and hair of Saudi Arabia smokers using modern analytical techniques. Int J Anal Chem. (2019) 2019:7125210. 10.1155/2019/7125210 31275390 PMC6582886

[33] Mohamed H Haris PI Brima EI. Estimated dietary intake of essential elements from four selected staple foods in Najran City, Saudi Arabia. BMC Chem. (2019) 13(1):73. 10.1186/s13065-019-0588-5 31384820 PMC6661740

[34] Kim S Seo YA. *In vitro* studies of manganese transport and homeostasis. Methods Enzymol. (2023) 687:185–206. 10.1016/bs.mie.2023.04.023 37666632

[35] France Štiglic A Falnoga I Briški AS Žavbi M Osredkar J Skitek M Reference intervals of 24 trace elements in blood, plasma and erythrocytes for the Slovenian adult population. Clin Chem Lab Med. (2024) 62(5):946–57. 10.1515/cclm-2023-0731 38008765

[36] Król E Bogdański P Suliburska J Krejpcio Z. The relationship between dietary, serum and hair levels of minerals (Fe, Zn, Cu) and glucose metabolism indices in obese type 2 diabetic patients. Biol Trace Elem Res. (2019) 189(1):34–44. 10.1007/s12011-018-1470-3 30091069 PMC6443611

[37] Kiani AK Dhuli K Donato K Aquilanti B Velluti V Matera G Main nutritional deficiencies. J Prev Med Hyg. (2022) 63(2 Suppl 3):E93. 10.15167/2421-4248/jpmh2022.63.2S3.275 36479498 PMC9710417

[38] Ekun O Salau I Madu N. Biochemical assessment of some trace elements in hypertensive patients. Pan Afr J Life Sci. (2021) 5:173–80. 10.36108/pajols/1202/50.0110

[39] Farooq D Alamri A Alwhahabi B Metwally A Kareem K. The status of zinc in type 2 diabetic patients and its association with glycemic control. J Family Community Med. (2020) 27(1):29–36. 10.4103/jfcm.JFCM_113_19 32030076 PMC6984028

[40] Toblli JE Gennaro FD Rivas C. Changes in echocardiographic parameters in iron deficiency patients with heart failure and chronic kidney disease treated with intravenous iron. Heart Lung Circ. (2015) 24(7):686–95. 10.1016/j.hlc.2014.12.161 25666998

[41] Dong F Zhang X Culver B Chew H Kelley R Ren J. Dietary iron deficiency induces ventricular dilation, mitochondrial ultrastructural aberrations and cytochrome c release: involvement of nitric oxide synthase and protein tyrosine nitration. Clin Sci. (2005) 109(3):277–86. 10.1042/CS20040278 15877545

[42] Naito Y Tsujino T Matsumoto M Sakoda T Ohyanagi M Masuyama T. Adaptive response of the heart to long-term anemia induced by iron deficiency. Am J Physiol Heart Circ Physiol. (2009) 296(3):H585–93. 10.1152/ajpheart.00463.2008 19136608

[43] Liu Y Zhao X Zhang X Zhao X Liu Y Liu J. Effects of oral administration of CrCl 3 on the contents of Ca, Mg, Mn, Fe, Cu, and Zn in the liver, kidney, and heart of chicken. Biol Trace Elem Res. (2016) 171:459–67. 10.1007/s12011-015-0559-1 26537118

[44] Szentmihályi K Vinkler P Fodor J Balla J Lakatos B. The role of manganese in the human organism. Orv Hetil. (2006) 147(42):2027–30. 17165602

[45] Choi M-K Bae Y-J. Relationship between dietary magnesium, manganese, and copper and metabolic syndrome risk in Korean adults: the Korea national health and nutrition examination survey (2007–2008). Biol Trace Elem Res. (2013) 156:56–66. 10.1007/s12011-013-9852-z 24218228

[46] Wang J Liu Q Shi W Cao L Deng R Pan T Synthesized manganese oxide nanorods: fabrication, characterization, application in cardiomyocyte protection from oxidative stress during sepsis, and evaluation of biochemical aspects of hemoglobin interaction. Arab J Chem. (2024) 17(10):105952. 10.1016/j.arabjc.2024.105952

[47] Xiao S Zhou Y Liu T Hu Y Wu Q Pan Q The association between manganese exposure with cardiovascular disease in older adults: NHANES 2011–2018. J Environ Sci Health Part A. (2021) 56(11):1221–7. 10.1080/10934529.2021.1973823 34474652

[48] Meishuo O Eshak ES Muraki I Cui R Shirai K Iso H Association between dietary manganese intake and mortality from cardiovascular disease in Japanese population: the Japan collaborative cohort study. J Atheroscler Thromb. (2022) 29(10):1432–47. 10.5551/jat.63195 35082202 PMC9529386

[49] Huang S Liu Z Ge X Luo X Zhou Y Li D Occupational exposure to manganese and risk of creatine kinase and creatine kinase-MB elevation among ferromanganese refinery workers. Am J Ind Med. (2020) 63(5):394–401. 10.1002/ajim.23097 32112445

[50] Li L Yang X. The essential element manganese, oxidative stress, and metabolic diseases: links and interactions. Oxid Med Cell Longev. (2018) 2018:7580707. 10.1155/2018/7580707 29849912 PMC5907490

[51] Mansouri B Azadi NA Sharafi K Nakhaee S. The effects of active and passive smoking on selected trace element levels in human milk. Sci Rep. (2023) 13(1):20756. 10.1038/s41598-023-48012-9 38007512 PMC10676413

[52] Tellez-Plaza M Jones MR Dominguez-Lucas A Guallar E Navas-Acien A. Cadmium exposure and clinical cardiovascular disease: a systematic review. Curr Atheroscler Rep. (2013) 15(10):356. 10.1007/s11883-013-0356-2 23955722 PMC3858820

[53] Moon S Lee J Yu JM Choi H Choi S Park J Association between environmental cadmium exposure and increased mortality in the U.S. national health and nutrition examination survey (1999–2018). J Expo Sci Environ Epidemiol. (2023) 33(6):874–82. 10.1038/s41370-023-00556-8 37161056

[54] Lin HC Hao WM Chu PH. Cadmium and cardiovascular disease: an overview of pathophysiology, epidemiology, therapy, and predictive value. Rev Port Cardiol (Engl Ed). (2021) 40(8):611–7. 10.1016/j.repc.2021.01.009 34392906

[55] Xu H Mao Y Xu B Hu Y. Low-level environmental lead and cadmium exposures and dyslipidemia in adults: findings from the NHANES 2005–2016. J Trace Elem Med Biol. (2021) 63:126651. 10.1016/j.jtemb.2020.126651 33035812

[56] Kim J Song H Lee J Kim YJ Chung HS Yu JM Smoking and passive smoking increases mortality through mediation effect of cadmium exposure in the United States. Sci Rep. (2023) 13(1):3878. 10.1038/s41598-023-30988-z 36890267 PMC9995499

[57] Mancusi C Basile C Spaccarotella C Gargiulo G Fucile I Paolillo S Novel strategies in diagnosing heart failure with preserved ejection fraction: a comprehensive literature review. High Blood Press Cardiovasc Prev. (2024) 31(2):127–40. 10.1007/s40292-024-00629-1 38489152 PMC11043114

[58] Omar AMS Bansal M Sengupta PP. Advances in echocardiographic imaging in heart failure with reduced and preserved ejection fraction. Circ Res. (2016) 119(2):357–74. 10.1161/CIRCRESAHA.116.309128 27390337

[59] Wechselberger C Messner B Bernhard D. The role of trace elements in cardiovascular diseases. Toxics. (2023) 11(12):956. 10.3390/toxics11120956 38133357 PMC10747024

[60] Ferreira JP Metra M Mordi I Gregson J ter Maaten JM Tromp J Heart failure in the outpatient versus inpatient setting: findings from the BIOSTAT-CHF study. Eur J Heart Fail. (2019) 21(1):112–20. 10.1002/ejhf.1323 30338883

